# cAMP-CRP-activated *E. coli* causes growth arrest under stress conditions

**DOI:** 10.3389/fmicb.2025.1597530

**Published:** 2025-08-29

**Authors:** Most Farzana Haque, Takefusa Tarusawa, Chisato Ushida, Shion Ito, Hyouta Himeno

**Affiliations:** ^1^Department of Biochemistry and Molecular Biology, Faculty of Agriculture and Life Science, Hirosaki University, Hirosaki, Japan; ^2^The United Graduate School of Agricultural Sciences, Iwate University, Morioka, Japan

**Keywords:** growth arrest, cAMP, salt stress, oxidative stress, *E. coli*, CRP

## Abstract

Cells are exposed to various kinds of stress, obliging them to cope with the challenges they encounter. Upon subjecting *E. coli* cells to oxidative stress induced by elevated concentrations of hydrogen peroxide or plumbagin, as well as osmotic stress by elevated NaCl concentrations, cell growth stopped immediately after stress and it resumed after a period of time. In the present study, we found that the stress-induced growth arrest disappeared when the gene for cAMP synthesis (*cyaA*) or its receptor protein (*crp*) was deleted, whereas it reappeared when cAMP is exogenously added to *∆cyaA* cells. With increasing stress intensity, the period of growth arrest increased in the wild-type cells, while *∆cyaA* or *∆crp* cells continued to grow without arrest, although their growth rates were reduced. These results indicate that *E. coli* cell has a novel cAMP-CRP-dependent mechanism to stop cell growth temporarily for adaptation to new environment when subjected to a strong stress, whether oxidative or osmotic stress.

## Introduction

Cells are often exposed to various kinds of stresses that threaten their survival. To cope with such emergencies, the cell is equipped with multiple stress response pathways. Although the bacterial stress response pathways have extensively been studied, only a few have focused on the growth arrest immediately following the stress. For example, glucose limitation or amino acid starvation causes temporary growth arrest of *E. coli* cells (Joseph et al., 1982; Kolb et al., 1993; Nystrom, 1997; Farewell et al., 1996; Potrykus and Cashel, 2008; Castaño-Cerezo et al., 2011; Meyer et al., 2021). During the arrested period, the gene expression pattern changes from normal gene expression to stress-responsive gene expression.

Growth arrest is also caused by osmotic stress. A sudden increase in salt concentration in the culture medium induces a rapid influx of potassium ions, dramatically altering solute concentrations within the bacterial cell. Simultaneously, cell growth immediately stops (Hase et al., 2009). Subsequently, the σ^70^-dependent general transcription switches to the σ^S^-specific transcription, allowing accumulation of osmoprotectants to alleviate abnormally high intracellular concentration of potassium ions (Rajkumari et al., 1996; Wood, 1999; Lee and Gralla, 2004), and then cell growth resumes after a certain period of time. We have shown that the arrested period is shortened by some kinds of manipulations, e.g., an impaired ribosome function due to a protein synthesis inhibitor, depletion of a ribosome maturation factor, depletion of a ribosomal protein (Hase et al., 2009, 2013) or increase in cellular concentration of the alarmone (p) ppGpp, which is involved in stringent response upon amino acid starvation (Tarusawa et al., 2016). However, questions remained as to why and how these manipulations lead to shortening of the arrested period. In the present study, we found that when the genes for adenylate cyclase (CyaA), which catalyzes the synthesis of cAMP, or cAMP receptor (CRP) were deleted, growth arrest was no longer observed in *E. coli* cells exposed to high levels of salt stress. Growth arrest, depending on cAMP and CRP, was also observed when cells were exposed to high levels of oxidative stress induced by either peroxide or superoxide. Given the limited overlap between osmotic and oxidative stress response pathways, there may be an as yet unknown mechanism behind the present finding.

Initially, cAMP was found to be induced upon glucose-limiting conditions in which CyaA is activated to produce cAMP (Kolb et al., 1993). It binds CRP to form cAMP-CRP complex, which regulates transcription of the genes specific for catabolism of alternative carbon sources. Later, it was found to be more widely involved in various kinds of cellular functions, such as flagellum biosynthesis (Bertin et al., 2014), biofilm formation (Jackson et al., 2002), virulence (Båga et al., 1985) and persistence (Molina-Quiroz et al., 2018). A recent study has suggested hundreds of promoter sequences on the *E. coli* genome involved in regulation by cAMP-CRP complex (Shimada et al., 2011). cAMP is also reported to be involved in stress-responsive events including osmotic stress (Landis et al., 1999) and oxidative stress (Barth et al., 2009) responses. Because cAMP-CRP functions as a transcription factor, cAMP-dependent gene expression is likely a time-consuming event that involves transcription, translation and product accumulation before exerting its effect. In contrast, the growth arrest that we focus on is a quick response that occurs immediately after stress. It might be a kind of defense system prepared in advance to be able to respond immediately to an emergency, rather than a system that begins to be built when an emergency occurs. Thus, the present study suggests the existence of a novel stress response system in bacterial cells that is distinct from known mechanisms and provides an example of a new type of adaptation strategy, while also revealing a novel function for cAMP.

## Materials and methods

### Bacterial strains

In this study, MG1655 (K-12) was used as the wild-type strain. Two mutants, *∆cyaA* (*cyaA*: FRT) and *∆crp* (*crp*: FRT), were constructed from Keio collection strains using P1 transduction (Baba et al., 2006). MG1655*∆rpoS* (*rpoS*: FRT) was described previously (Tarusawa et al., 2016). The expected rearrangement of the genome was confirmed by PCR.

### Culture conditions, stress method and OD monitoring

All strains were precultured in LB medium for 15–16 h at 37°C, 125 rpm. They were transferred into a total of 9 mL LB media using the 18 mm tube to give a final OD_600_ value of 0.005, maintaining temperature and shaking is 37°C and 180 rpm, respectively (Supplementary Figure S1). When OD_600_ reached 0.7, 3 mL of NaCl/H_2_O_2_/plumbagin was added to the culture medium. OD_600_ values were automatically recorded at 1 min intervals using a non-invasive turbidity meter (TAITEC Co., Saitama, Japan). When OD_600_ = 2.5 or higher, it exceeds the limit of the turbidity meter, so an appropriate amount of the culture solution was taken out at a fixed time, diluted 10 times, and then measured manually using a spectrophotometer.

### Exogenous addition of cAMP

cAMP powder (Nacalai Tesque, Inc., Kyoto, Japan) was dissolved into H_2_O according to the manufacturer’s protocol. A 10 mM (final concentration) cAMP was used for NaCl or H_2_O_2_ stress, and 3 mM cAMP was used for plumbagin stress. cAMP was added to the media at OD_600_ = 0, 0.3, 0.5 or 0.7, while 0.9 M NaCl, 70 mM H_2_O_2_ or 20 μg/mL plumbagin was added at OD_600_ = 0.7.

### Measurement of reactive oxygen species (ROS)

Reactive oxygen species was measured under salt and oxidative stress conditions. One milliliter of culture was collected immediately before and 1, 2, 4, 7, 10, 15, 30 and 60 min after stress introduced at OD_600_ = 0.7. Thereafter, the cells were incubated at 95°C for 5 min. Cells were washed with PBS buffer (137 mM NaCl, 2.7 mM KCl, 10 mM Na_2_HPO_4_ and 1.8 mM KH_2_PO_4_) 3 times and suspended in the PBS buffer. Thirty micromolar (final concentration) of H_2_DCFDA (2′,7′-dichlorodihydrofluorescein diacetate) dye (Invitrogen, Thermo Fisher Scientific, Waltham, MA, United States) was added to the PBS buffer and incubated the cells at 37°C for 30 min. After incubation cells were washed with PBS buffer 3 times and suspended again in PBS buffer. The absorbance was measured by a fluorometer (Thermo Fisher Scientific) with an excitation wavelength of 488 nm and an emission wavelength of 525 nm.

### Measurement of cAMP concentration

One milliliter of cell culture was harvested immediately before and indicated time points after stress introduced at OD_600_ = 0.7. It was centrifuged at 12,000 *g* for 2 min at 4°C and the supernatant was used for measurement of extracellular cAMP concentration (Inada et al., 1996). The pellet was suspended in 20 μL of H_2_O and heated for 5 min at 95°C, and then it was centrifuged at 12,000 *g* for 2 min at 4°C. Supernatant was mixed with 60 μL ethanol, then centrifuged at 12,000 *g* for 10 min at 4°C. The supernatants were dried, and pellets (pellets of cAMP) were suspended in 10 μL of H_2_O and used for the determination of intracellular cAMP levels (Inada et al., 1996).

Cyclic AMP Select ELISA Kit (Cayman Chemical, Ann Harbor, MI, United States) was used to measure the intracellular and extracellular cAMP concentration. Experimental samples and standard samples included in the kit were loaded into the 96-well plates according to the manufacturer’s protocol, then incubated at 4°C for 18 h and washed with ELISA washing buffer. Freshly prepared Ellman’s reagent was added to all wells and was shaken on an orbital shaker at 500 rpm for 90–120 min. A multi-spectrophotometer, (VIENTO, Dainippon Sumitomo Pharma, Co., Ltd., Osaka, Japan) was used to record the absorbance. The calibration curve was generated by plotting the absorbance values of the control standards against their corresponding cAMP concentrations using a semi-logarithmic scale (logarithmic *x*-axis for concentration and linear *y*-axis for absorbance). A four-parameter logistic (4PL) model was fitted to the data using the ELISADoulble program, yielding the equation: *y* = variant – ln (*x*) – variant where y represents the cAMP concentration and x represents the sample absorbance under each condition.

## Results

### Salt stress induces growth arrest depending on cAMP and CRP

**Figure 1:**
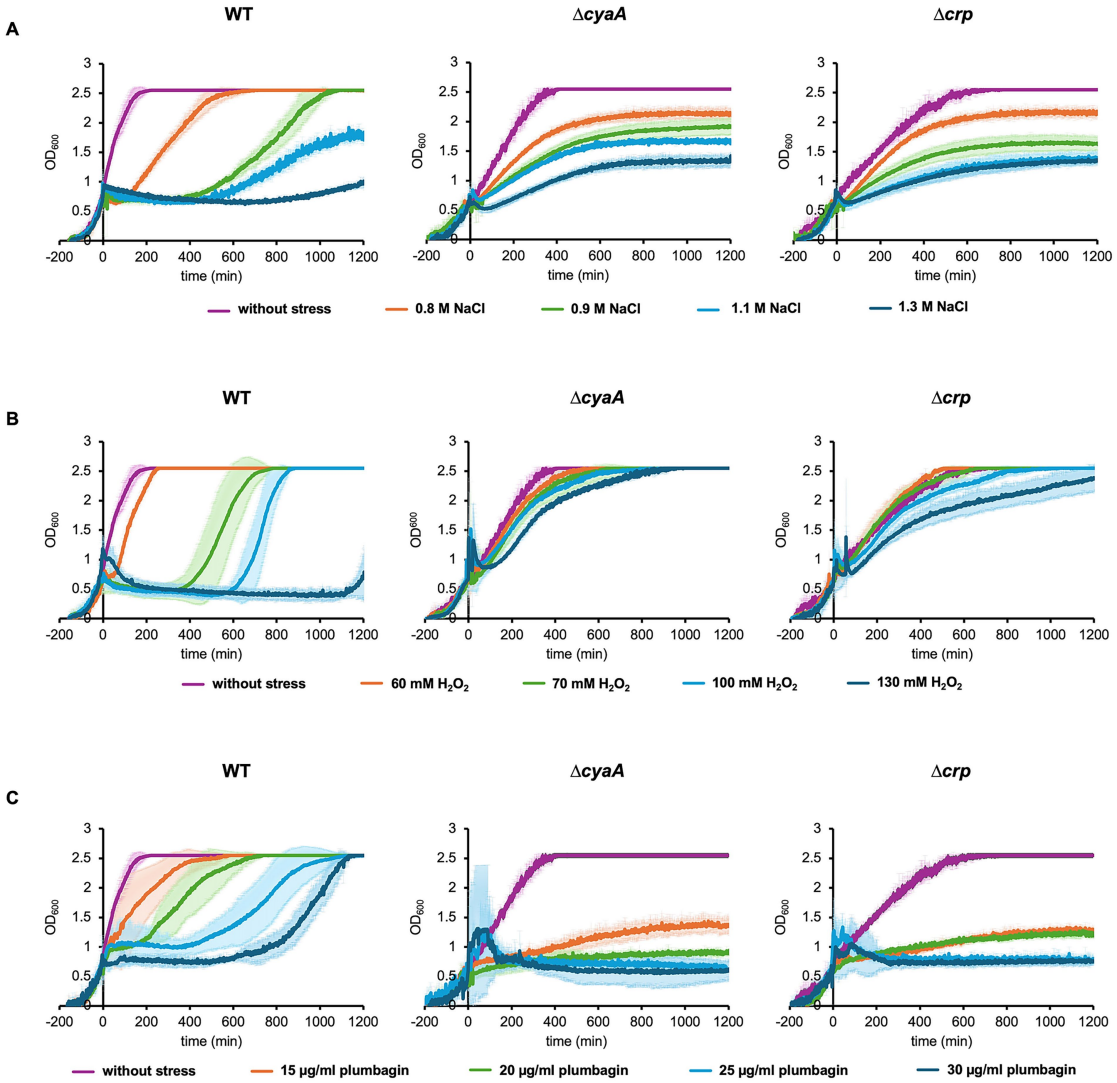
Growth of *E. coli* cells with and without stress. Growths of wild-type, *∆cyaA* and *∆crp* cells were monitored with the addition of 0–1.3 M NaCl **(A)**, 0–130 mM H_2_O_2_ **(B)**, and 0–30 μg/mL plumbagin **(C)** at OD_600_ = 0.7. At least three independent experiments were performed for each culture condition. Error bars represent the standard deviation.

Through our previous studies, we have noticed that elevated concentrations of salt causes growth arrest of *E. coli* cells and that the arrested period is shortened by addition of a protein synthesis inhibitor, depletion of a ribosome maturation factor or depletion of a ribosomal protein (Hase et al., 2009, 2013). During the course of this line of research, we found that growth arrest did not occur in cells lacking the enzyme of cAMP synthesis or cAMP receptor. We monitored the time course of growth of wild-type, *∆cyaA* and *∆crp* cells automatically using a turbidity meter capable of recording the OD_600_ values up to 2.55. Under normal conditions, wild-type cells grew faster than *∆cyaA* or *∆crp* cells (Figure 1A). This result seems plausible given that bacterial cells are unable to properly regulate the metabolic pathways in the absence of CRP or cAMP, resulting in reduced metabolic flux and slower growth (Wehrens et al., 2023). As shown previously (Hase et al., 2009, 2013), addition of 0.9 M NaCl to the growth medium at OD_600_ = 0.7 caused the wild-type cells to pause growth and then resume growth several hours later. The higher the salt concentration, the longer the arrest time. At 0.8 M NaCl, the arrest time was too short to detect the arrest itself. Interestingly, the *∆cyaA* and *∆crp* cells did not show growth arrest after the addition of 0.9 M NaCl and continued to grow. Essentially the same results were obtained when salt stress is induced at OD_600_ = 0.5 (Supplementary Figure S2). Either *∆cyaA* or *∆crp* cells continued to grow without arrest even when the salt concentration was increased, but the growth rate was reduced (Figure 1A). These results suggest that both CyaA and CRP are responsible for growth arrest induced by salt stress. We then measured colony forming units (CFU) for about 600 min after stress was introduced (Supplementary Figure S3). For all three cell types, the time course of CFU appeared to roughly reflect the time course of OD_600_, suggesting that the majority of cells remained viable despite salt stress.

We also found that after recovery from arrest, wild-type cells showed better growth than *∆cyaA* or *∆crp* cells. Since the OD_600_ value exceeded the measurement limit of the automatic turbidity meter, we manually measured the cell growth during the stationary phase after salt stress (Supplementary Figure S4). CFU was also measured during this phase. The levels of growth as well as CFU at 24–32 h of *∆cyaA* or *∆crp* cells after salt stress were apparently lower than those of wild-type cells. This indicates that ∆*cyaA* or ∆*crp* cells continue to grow without arrest, and they are subsequently overtaken by wild type cells that undergo growth arrest for several hours after stress, which is also partially shown in Figure 1.

### Oxidative stress also induces growth arrest depending on cAMP and CRP

We next focused on oxidative stress to examine whether the growth arrest is specific to osmotic stress or also caused by other types of stress. There are two types of reactive oxygen species, peroxide and superoxide, each of which has its own specific oxidative stress response system (Chiang and Schellhorn, 2012; Pomposiello and Demple, 2001). It has been reported that the *∆cyaA* and the *∆crp* mutants are sensitive to superoxide radicals (methyl viologen, plumbagin) and highly resistant to peroxide and acid stress (Barth et al., 2009; Donovan et al., 2013). We introduced an oxidative stress with H_2_O_2_ as the peroxide and plumbagin as the superoxide. When we added 70 mM H_2_O_2_ at OD_600_ = 0.7, only wild-type cells showed growth arrest, whereas the *∆cyaA* mutant and the *∆crp* cells did not (Figure 1B). Essentially the same results were obtained when plumbagin was added instead of H_2_O_2_ as a stress inducer (Figure 1C). The growth arrest observed only in the wild-type occurred equally whether oxidative stress was added at OD_600_ = 0.7 or OD_600_ = 0.5 during oxidative stress (Supplementary Figure S2). The duration of growth arrest is also dose-dependent. Increasing either H_2_O_2_ or plumbagin concentration added increased the duration of growth arrest (Figures 1B,C). Taken together, these results indicated that salt stress and oxidative stress due to peroxide and superoxide all induce growth arrest in the wild-type cells but not *∆cyaA* and *∆crp* cells (Figure 1). We then measured CFU after oxidative stress was introduced (Supplementary Figure S4). When stressed with plumbagin, the CFUs seemed to roughly reflect cell proliferation, which was similar to that observed when stressed with NaCl. On the other hand, the CFU of wild-type cells was decreased when the cells were stressed with H_2_O_2_, which was slightly different from the results when the cells were stressed with NaCl or plumbagin. It can be accounted for by the number of connected cells: Microscopic observation showed that the number of connected cells increased when stressed with H_2_O_2_ but did not when stressed with salt or plumbagin (Supplementary Figure S5).

As with salt stress, after recovery from arrest, wild-type cells showed better growth than *∆cyaA* or *∆crp* cells (Figure 1). In addition, the growth level of ∆*cyaA* or ∆*crp* cells at 24–32 h after oxidative stress is lower than that of wild-type cells (Supplementary Figure S4). The difference was most pronounced when stress was induced by plumbagin. CFU of ∆*cyaA* or ∆*crp* cells at 24–32 h after oxidative stress is also lower than that of wild-type cells. Taken together, ∆*cyaA* or ∆*crp* cells continued to grow without arrest, and were then overtaken by wild-type cells that underwent growth arrest for several hours after stress.

### Stress-induced growth arrest reappeared when cAMP is exogenously added to *cyaA* cells

**Figure 2:**
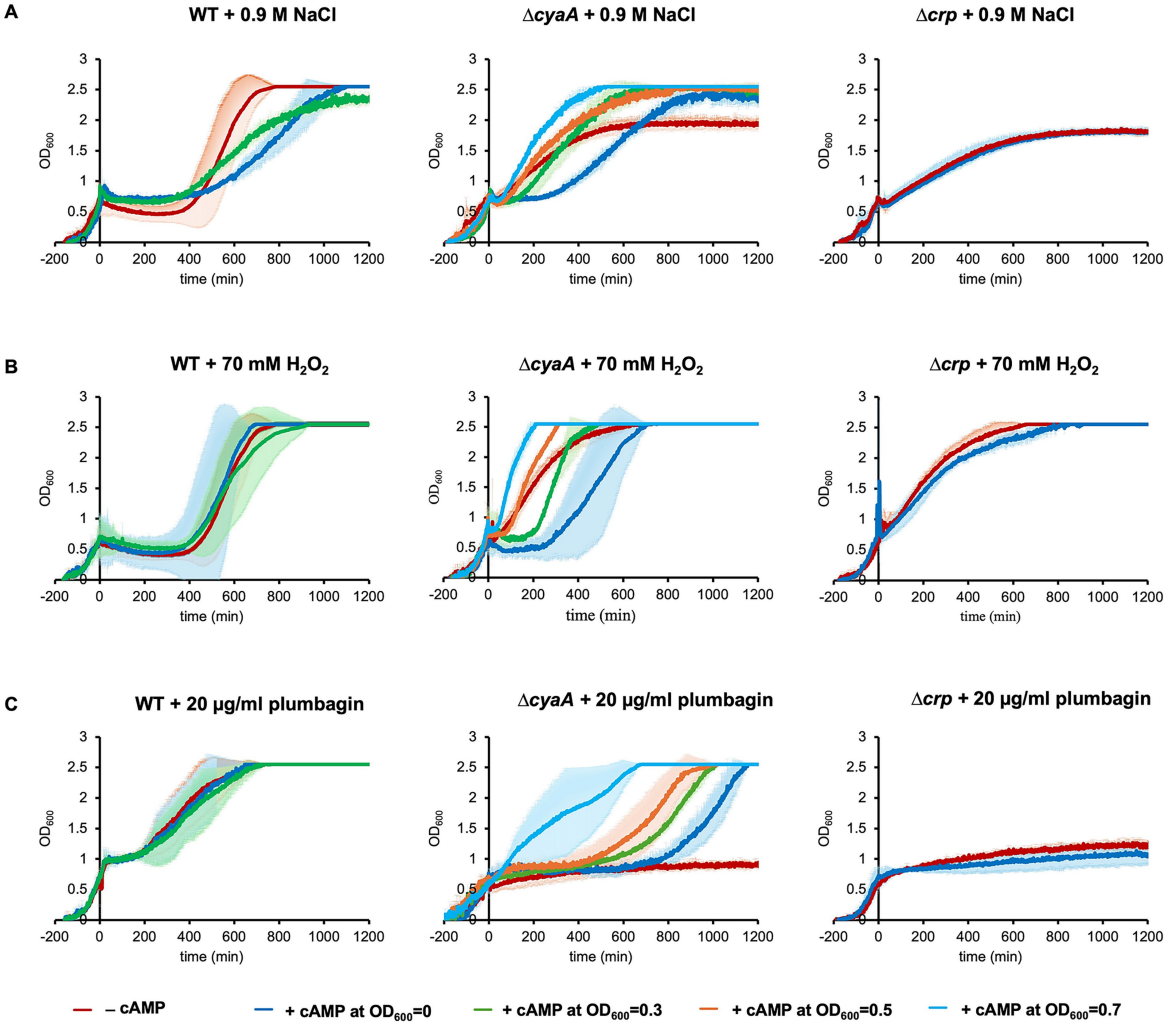
Exogenous addition of cAMP prior to stress. Growths of wild-type, *∆cyaA* and *∆crp* cells were monitored with the exogenous addition of 10 mM of cAMP at OD_600_ = 0, 0.3, 0.5 or 0.7. 0.9 M of NaCl **(A)**, 70 mM of H_2_O_2_ **(B)** or 20 μg/mL plumbagin **(C)** was added at OD_600_ = 0.7 to introduce stress. At least three independent experiments were performed for each culture condition. Error bars represent the standard deviation.

It has been shown that addition of exogenous cAMP to the medium abolishes the typical diauxic lag (Ullmann and Monod, 1968). This indicates that exogenous cAMP is incorporated into cells to exert its effects and therefore we added cAMP to the medium before stress was introduced (Figure 2). As expected, addition of exogenous cAMP caused the *∆cyaA* cells to undergo growth arrest upon salt stress (Figure 2A). In contrast, addition of exogenous cAMP had no effects on the growth of *∆crp* cells, indicating that both cAMP and CRP are required for growth arrest. This result makes sense, considering that cAMP rarely acts alone but most often acts in complex with CRP.

We also found that the timing of exogenous cAMP addition was related to its effect. Usually, we exogenously added cAMP at the start of culture to arrest growth. When exogenous addition of 10 mM cAMP to the culture medium was performed immediately before salt stress introduced at OD_600_ = 0.7, *∆cyaA* cells no longer underwent growth arrest (Figure 2A). The earlier the timing of exogenous cAMP addition, the longer the duration of growth arrest. Similar results were obtained when H_2_O_2_ or plumbagin was used instead of NaCl (Figures 2B,C). These results indicate that cAMP needs time to function in stress-induced growth arrest irrespective of the type of stress.

We investigated the effect of adding lower concentrations of exogenous cAMP to ∆*cyaA* cells (Supplementary Figure S6). When 3 mM or 5 mM cAMP was added exogenously, growth arrest due to NaCl or H_2_O_2_ stress was hardly observed. Growth arrest due to plumbagin stress was still observed, but the period of growth arrest was shortened in a cAMP concentration-dependent manner. Collectively, the period of stress-induced growth arrest was shortened by decreasing the exogenous cAMP concentration.

### Intracellular cAMP level increased immediately after stress

**Figure 3:**
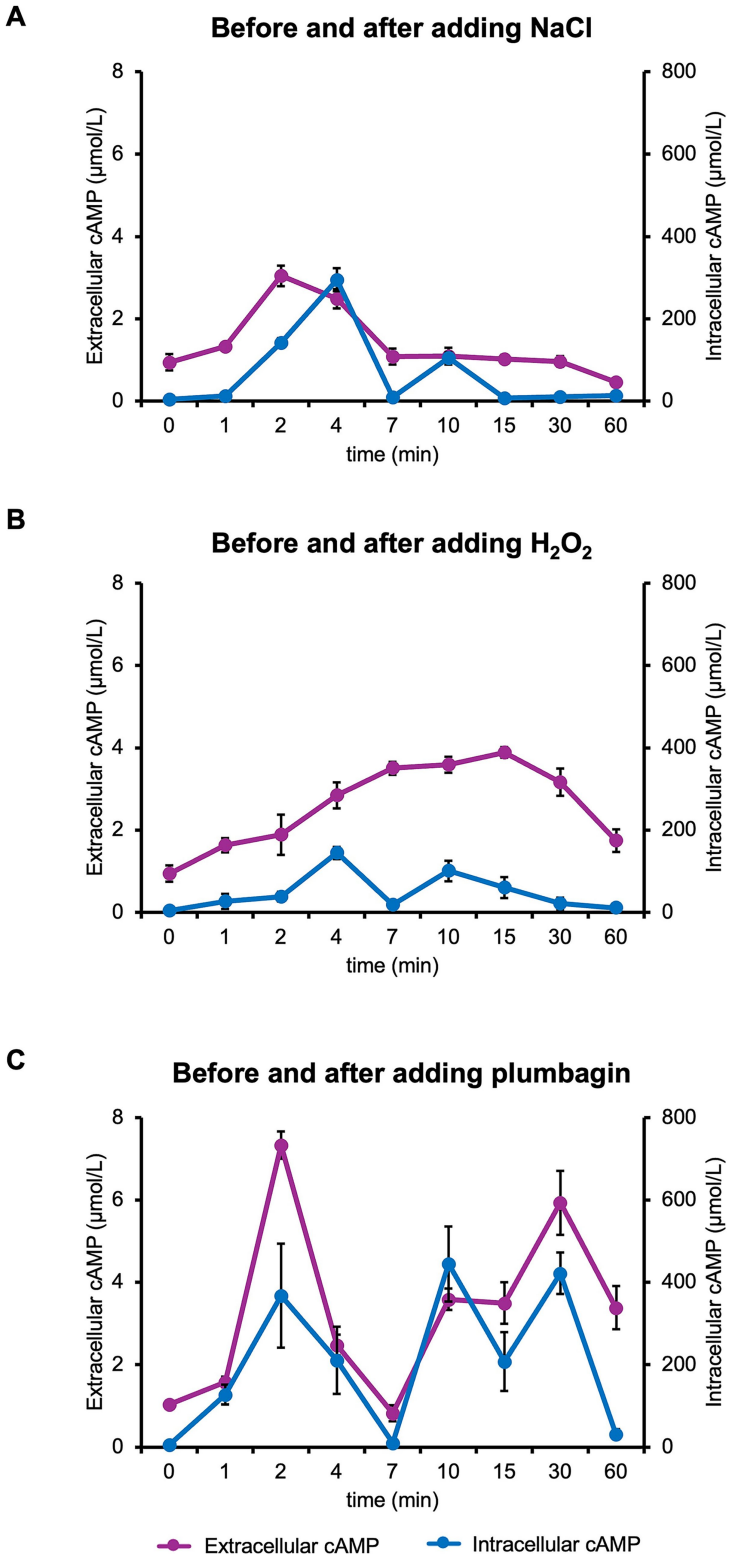
Intracellular and extracellular cAMP levels after stress. cAMP level in wild-type cells was measured at the indicated time points just before (0 min) and 1–60 min after the addition of NaCl **(A)**, H_2_O_2_ **(B)** or plumbagin **(C)** at OD_600_ = 0.7, was measured. At least three independent experiments were performed for each culture condition. Error bars represent the standard deviation.

It has been shown that the cellular cAMP regulation changes in response to stress introduced by environmental stimuli or antibiotic treatment (Nosho et al., 2018; Barth et al., 2009; Cheung et al., 2009). We measured intracellular cAMP levels after salt stress, focusing on the period immediately following stress, specifically from just before stress onset to 60 min afterward. We found that intracellular cAMP level increased significantly within a few minutes after salt stress and then decreased to basal level by 60 min (Figure 3A). Similar rapid increases of intracellular cAMP concentration were also observed when H₂O₂ or plumbagin was used as a stress inducer (Figures 3B,C). ∆*cyaA* cells lacking the enzymes required for endogenous cAMP synthesis are unlikely to undergo a rapid increase in cAMP immediately after stress, even when exogenous cAMP is added. However, Δ*cyaA* cells exhibit growth arrest similar to that of wild-type cells. These observations suggest that the rapid increase in cAMP immediately after stress is not required for stress-induced growth arrest.

### Effect of adding glucose to the medium

**Figure 4:**
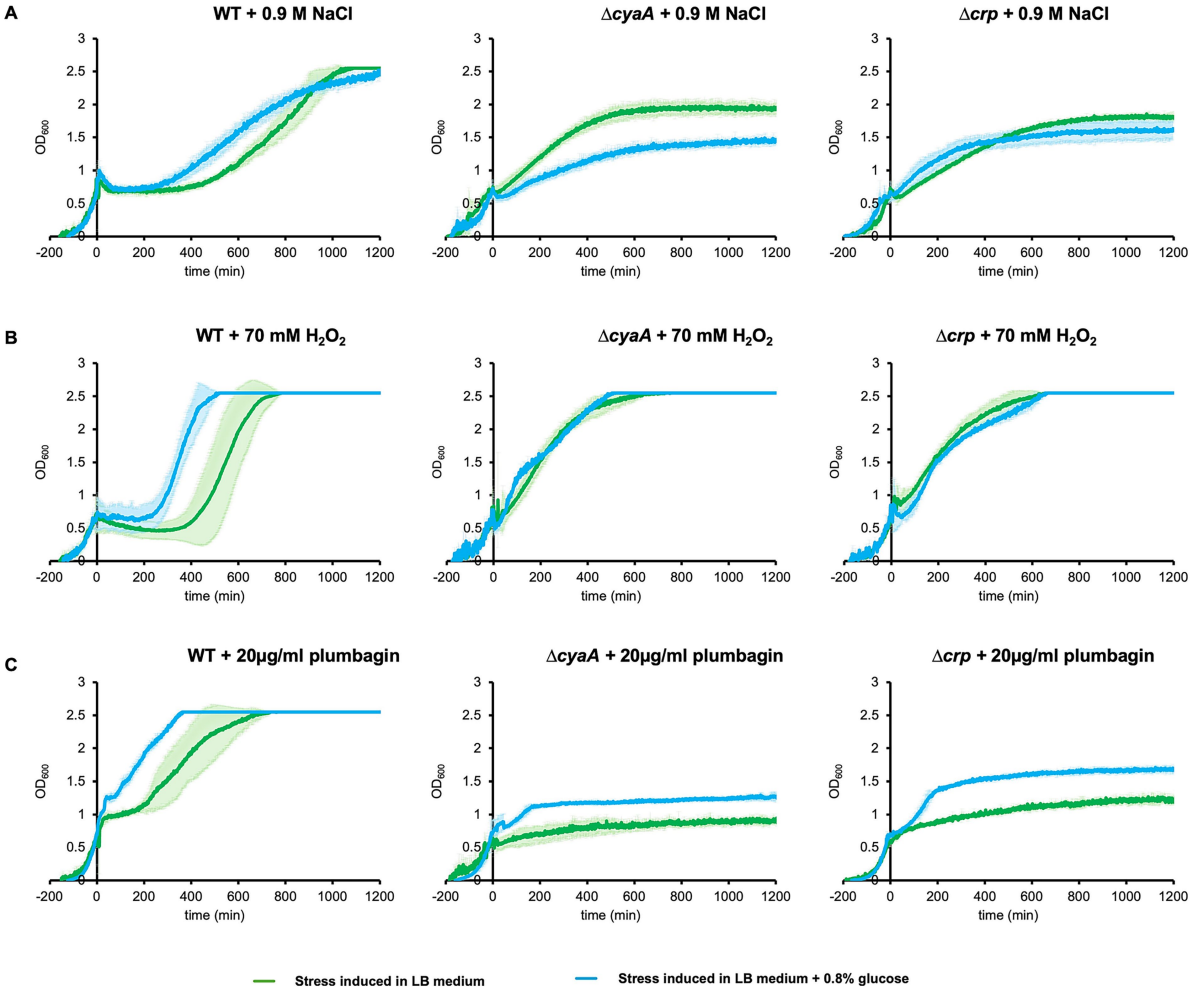
Effect of addition of glucose to the medium on stress-induced growth arrest. Wild-type, *∆cyaA* and *∆crp* cells were cultured in the LB medium supplemented with 0.8% glucose and stressed at OD_600_ = 0.7 by adding 0.9 M NaCl **(A)**, 70 mM H_2_O_2_ **(B)**, and 20 μg/mL plumbagin **(C)**, and growth was measured. At least three independent experiments were performed for each culture condition. Error bars represent the standard deviation.

Addition of glucose to the medium reduces the intracellular cAMP concentration in *E. coli* (Epstein et al., 1975), and this was confirmed under our experimental conditions. Then, we investigated its effect on stress-induced growth arrest. As expected, addition of 0.8% glucose to the medium decreased the duration of arrest period upon stress induced by salt, H_2_O_2_ and plumbagin (Figure 4). This is consistent with the above results showing that cAMP is responsible for stress-induced growth arrest.

### Intracellular ROS is not responsible for growth arrest

**Figure 5:**
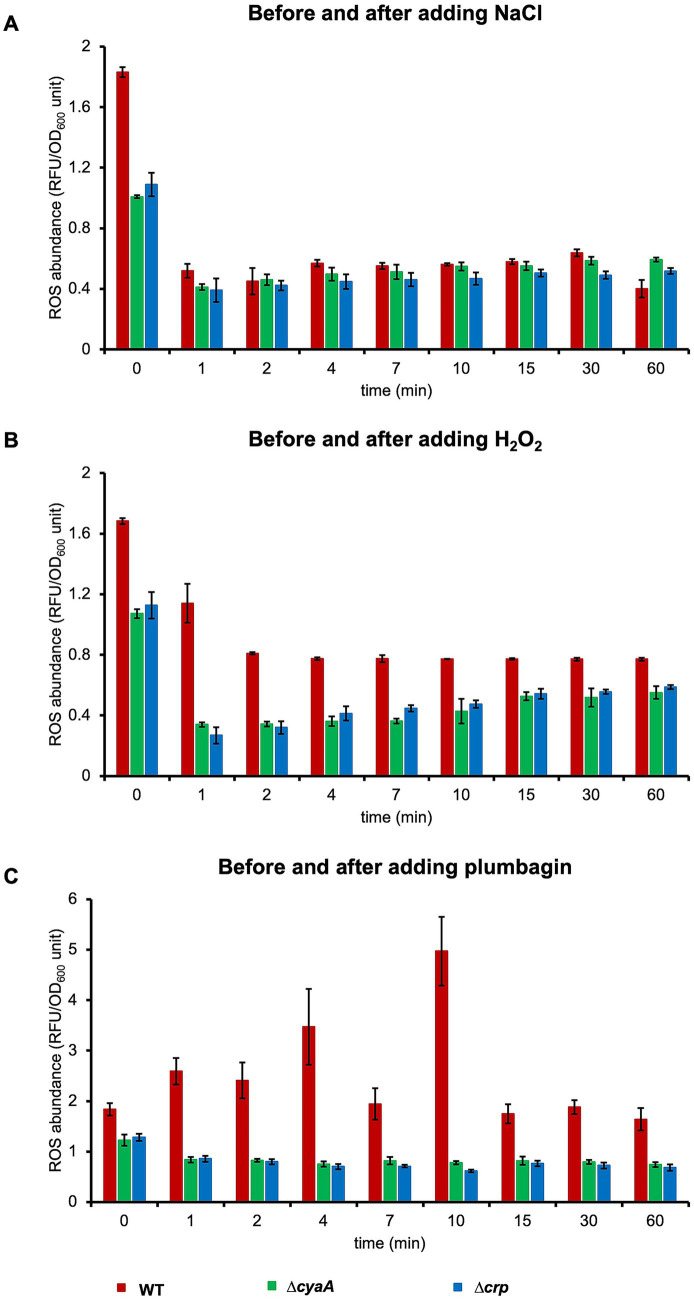
Intracellular ROS levels after stress. ROS levels in the cells were measured at the indicated time points just before (0 min) and 1–60 min after the addition of NaCl **(A)**, H_2_O_2_ **(B)** or plumbagin **(C)** at OD_600_ = 0.7. At least three independent experiments were performed for each culture condition. Error bars represent the standard deviation.

Oxidative stress triggers production of reactive oxygen species (ROS), leading to bacterial cell death (Touati, 2000). To examine the involvement of ROS in growth arrest, we measured the intracellular ROS immediately after salt or oxidative stress using a fluorometer. Before stress, the level of ROS in *∆cyaA* or *∆crp* cells (1.1 RFU/OD unit) is a little lower than that in wild-type cells (1.8 RFU/OD unit). The intracellular level of ROS in all of these three strains was reduced within 1 min after salt stress, but the decrease then stopped and remained at similar levels in all three strains by 60 min (Figure 5A). When oxidative stress was applied with H_2_O_2_, a similar time course of ROS levels was observed, although they remained slightly lower in *∆cyaA* or *∆crp* cells than in the wild-type cells from 1 to 60 min after oxidative stress (Figure 5B). Plumbagin-induced oxidative stress showed a slightly different time course: intracellular ROS levels showed a slight increase or little change with fluctuation in wild-type cells, while they decreased very slowly in ∆*cyaA* and ∆*crp* cells (Figure 5C). Collectively, no significant trends were observed among the three types of stress with regard to ROS generation, and therefore ROS generation is unlikely to be involved in stress-induced growth arrest.

### The general stress-responsive transcription factor, σ^S^, is involved in the cell growth during later stages after stress rather than stress-induced growth arrest

**Figure 6:**
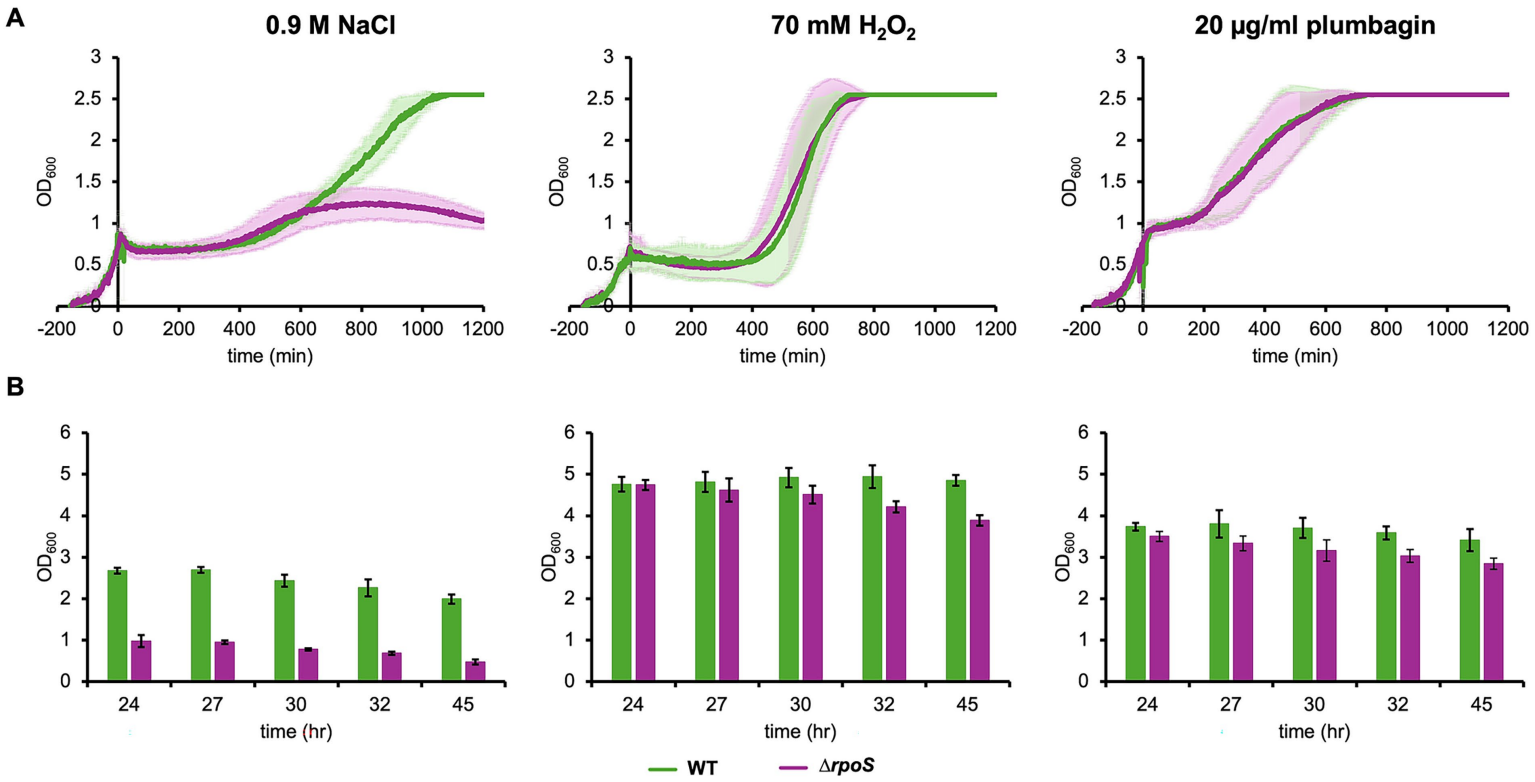
Growth of *∆rpoS* cells under salt and oxidative stresses. Growth of *∆rpoS* cells with the addition of 70 mM H_2_O_2_, 20 μg/mL plumbagin or 0.9 M NaCl at OD_600_ = 0.7 was monitored by a turbidity meter **(A)** and manually **(B)**. At least three independent experiments were performed for each culture condition. Error bars represent the standard deviation.

An alternative sigma factor, σ^S^, encoded by the *rpoS* gene, functions in response to multiple stress conditions, including osmotic and oxidative stresses (Bouillet et al., 2024). We have already shown that growth arrest induced by salt stress still occurs even in the *E. coli* cells lacking *rpoS*, *∆rpoS* cells (Tarusawa et al., 2016). In the present study, we confirmed that growth arrest occurred in ∆*rpoS* cells after salt stress (Figure 6A). The effect of deletion of *rpoS* appeared at a later stage. Specifically, the level of cell growth in the stationary phase significantly reduced. Growth arrest was also observed even after oxidative stress with either H_2_O_2_ or plumbagin. In addition, reduction in the level of cell growth in the stationary phase was also observed upon oxidative stress with either H_2_O_2_ or plumbagin (Figure 6B). These results indicate that σ^S^ is not involved in stress-induced growth arrest and that it is involved in cell growth at a later stage after stress, especially salt stress.

## Discussion

In the present study, we demonstrated that *E. coli* cells possess a mechanism to rapidly arrest cell growth upon exposure to strong stress, whether osmotic or oxidative stress, to adapt to the new environment, which requires cAMP-CRP before the stress occurs. This finding suggests the existence of a novel stress response system with a novel function of cAMP in bacterial cells and thus represents an example of a new type of adaptation strategy, which allows focusing on stress-responsive gene expression by minimizing normal cellular activities until stress resistance is conferred. It is considered to be a kind of defense system prepared in advance to respond immediately to an emergency, rather than a system that begins to be built when an emergency occurs.

The wide variety of stress response pathways have been identified in bacteria, and they vary depending on the type of stress. Salt stress triggers water efflux from *E. coli* cell, inducing cell shrinkage, which are sensed by osmosensors embedded in the cytoplasmic membrane (Wood, 1999). It promotes rapid influx of potassium ions through the transporters to counteract the osmotic pressure (McLaggan et al., 1994), which then switches the σ^70^-dependent general transcription to σ^S^-specific transcription (Rajkumari et al., 1996; Lee and Gralla, 2004). *E. coli* cells have another osmotic stress response pathway controlled by σ^E^, an alternative sigma factor that is involved in expression of periplasmic and outer membrane proteins (Raivio and Silhavy, 2001; Alba and Gross, 2004). There are two types of reactive oxygen species, peroxide and superoxide, and there is a corresponding oxidative stress response system specific to each type. Peroxide stress induced by hydrogen peroxide is sensed by OxyR, activating the expression of downstream oxidative stress-responsive genes including the gene for catalase (Chiang and Schellhorn, 2012; Pomposiello and Demple, 2001). Superoxide stress induced by plumbagin is sensed by SoxR, which activates the transcription for superoxide dismutase (Pomposiello and Demple, 2001). The present study revealed that despite the diversity of stress-response pathways, cells have a potential to undergo growth arrest upon exposure to any of three different types of stress: osmotic stress, peroxide stress and superoxide stress.

In addition to the specific stress-responsive pathways, there is a general stress-responsive pathway governed by σ^S^. σ^S^ is an alternative sigma factor, acts as the stationary phase-specific transcription factor and also functions under many stress conditions, including osmotic stress described above. It accumulates in the cell due to increased osmolality (Muffler et al., 1996; Hengge-Aronis, 1996). It is also involved in oxidative stress in relation to the cellular cAMP level (Barth et al., 2009). σ^S^ significantly accumulated in the cells lacking *cyaA* or *crp*, and thus transcription of *rpoS*, the gene for σ^S^, has been proposed to be negatively regulated by cAMP-CRP complex (Lange and Hengge-Aronis, 1994). The intracellular level of σ^S^ under osmotic stress condition is regulated not only by increased transcription but also increased translation and reduced proteolysis (Muffler et al., 1996). Thus, σ^S^ often functions in general stress responses and is sometimes associated with cAMP-CRP. However, the present study demonstrated that stress-induced growth arrest still occurred in cells depleted of σ^S^ (Figure 6), indicating that σ^S^ is not involved in stress-induced growth arrest.

In this study, a rapid increase in intracellular cAMP concentration was observed immediately after stress (Figure 3). Since Δ*cyaA* cells cannot synthesize cAMP, it is unlikely that such a rapid increase in cAMP concentration would occur even if exogenous cAMP was added. Nevertheless, these cells still exhibited growth arrest. Therefore, a rapid increase in cAMP concentration immediately after stress is not necessarily required for stress-induced growth arrest. Notably, a rapid increase in intracellular cAMP concentration was observed under both salt and oxidative stress conditions. It may contribute in some way to the early phase of the cellular stress response.

**Figure 7:**
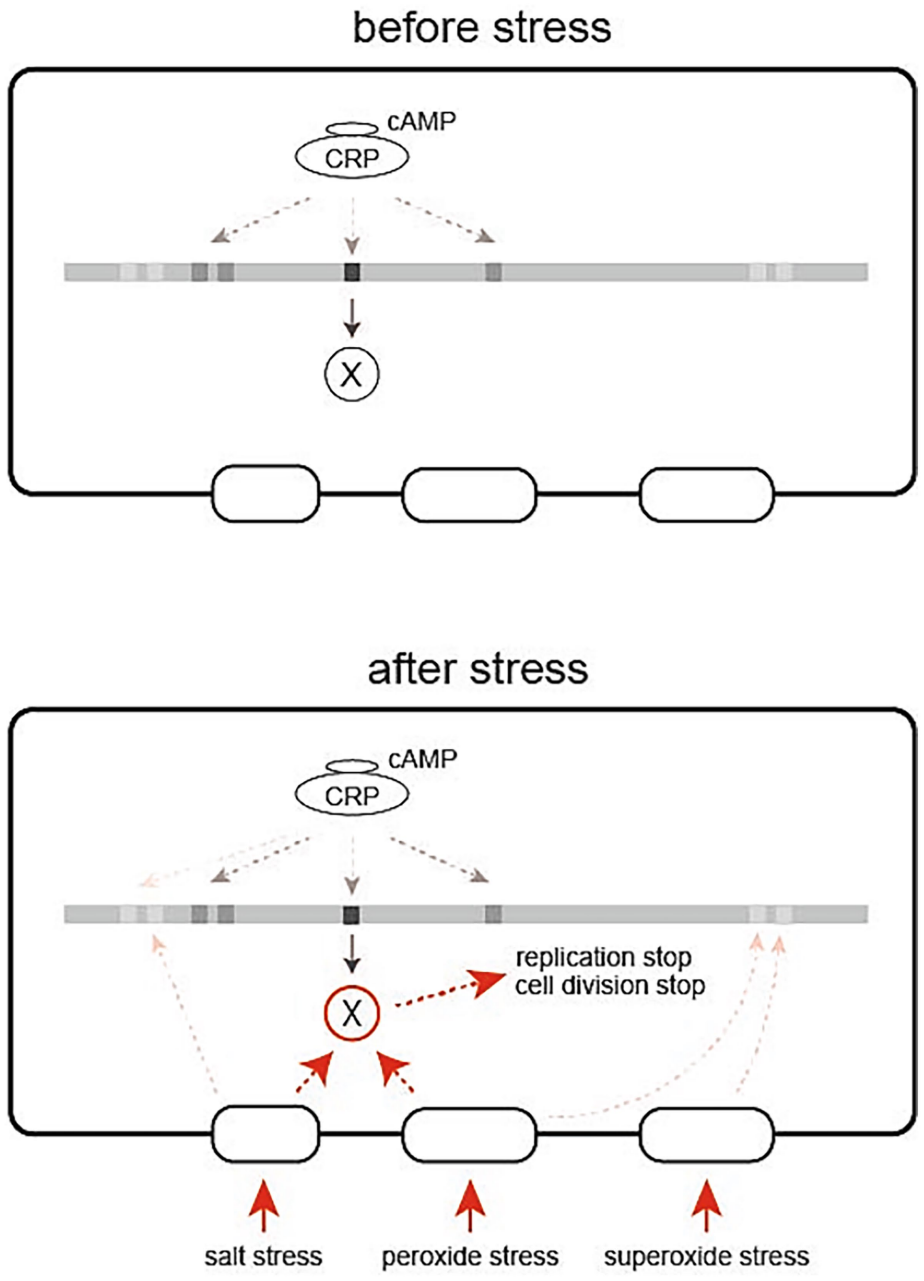
A model of stress-induced growth arrest of *E. coli* cells depending on cAMP-CRP. cAMP-CRP acts as a transcription factor to produce protein X, which is inactive until the cell is exposed to stress (left). When the cell is exposed to stress, protein X accumulated in the cell quickly becomes active to stop the fundamental cellular functions such as replication and cell division (right).

Besides the very limited overlap between osmotic and oxidative stress response pathways, lack of the significance of the general stress-responsive transcription factor σ^S^ for stress-induced growth arrest strongly suggests the presence of a yet unknown mechanism behind the present finding. Figure 7 shows a model of stress-induced growth arrest, which assumes protein X as a key member of the system, which is produced depending on cAMP-CRP and reaches sufficient levels before stress. It is possible, but unlikely, that protein X is a transcription factor, because growth arrest is a rapid response to stress and thus it is unlikely to involve transcription, translation and product accumulation. Therefore, it seems reasonable to assume a mediator molecule, i.e., protein X, that receives stress signals from the sensors and transmits them to molecules that quickly shut down most cellular activities, including DNA replication and cell division. In order to respond immediately to the stress, a certain amount of protein X needs to be produced before the stress occurs.

Then the question arises: how is the stress signal rapidly transmitted from the stress sensor to protein X and from protein X to downstream proteins? Conformational change, post-translational modification and protein degradation can be used as a means to rapidly transmit signals from a protein to the partner proteins. Considering that growth arrest occurs in response to at least three different stresses, salt stress, peroxide stress, and superoxide stress, there may be multiple types of protein Xs that correspond to different sensors.

Thus, the stress-induced growth arrest is considered as a defense system prepared in advance to respond immediately to an emergency, rather than a system that begins to be built when an emergency occurs. Although cAMP-depleted cells do not induce growth arrest upon salt or oxidative stress, growth arrest appears when cAMP is exogenously added. The duration of growth arrest depended on the amount of exogenous cAMP (Supplementary Figure S6) and the timing of its addition (Figure 2). Its addition just prior to the stress has little effect on growth arrest. The longer the time between exogenous addition of cAMP and stress, the longer the duration of growth arrest. These results also rationalize the assumption of protein X. cAMP requires time to exert its effect on growth arrest, a time likely required for transcription, translation and accumulation of protein X. The length of the span of growth arrest during which induction of stress-responsive gene expression proceeds might reflect the level of accumulated protein X. Even if protein X sufficiently accumulates within the cell, it would remain inactive until the cell is exposed to stress. Shortening of the salt-stress-induced arrest period has been observed with the addition of a protein synthesis inhibitor, depletion of a ribosome maturation factor, depletion of a ribosomal protein or upshift of (p)ppGpp (Hase et al., 2009, 2013; Tarusawa et al., 2016). Presumably, these manipulations could reduce the level of protein X accumulated through reduced translation or transcriptional modulation, thereby shortening the arrest period. The length of the period of growth arrest depends not only on the timing of addition of exogenous cAMP to cAMP-depleted cells probably indicating the cellular level of protein X but also on the intensity of stress. As stress intensity increased, the duration of growth arrest became longer in wild-type cells (Figure 1), probably reflecting an increasing requirement of expression of stress-responsive genes with stress intensity. This is in contrast to cAMP-depleted cells, where increasing stress intensity reduced the growth rate but did not result in growth arrest (Figure 1).

Growth of the wild-type cells was arrested immediately after stress but exceeded that of the non-arrested ∆*cyaA* or ∆*crp* cells after recovery of growth (Figure 1), and the full growth levels of the wild-type cells after the stress were higher than those of the ∆*cyaA* and ∆*crp* cells (Supplementary Figure S2). These results suggest that growth arrest immediately following stress has a positive effect on subsequent growth. Although stress-induced growth arrest may appear to have a negative impact in the short term, it can ultimately be considered a sophisticated stress adaptation strategy that expands the stress-tolerant population.

The importance of cAMP and CRP was first discovered under glucose-limiting condition (Kolb et al., 1993). In this condition, a temporary growth arrest is observed during the metabolic shift from glucose utilization to an alternative sugar (lactose) utilization. However, it apparently differed from growth arrest caused by salt or oxidative stress. Upon glucose limitation, the cAMP level is transiently increased during growth arrest (Inada et al., 1996), while no significant cAMP fluctuation was observed immediately after the salt or oxidative stress (Figure 3). Moreover, growth arrest under glucose limitation disappears by exogenous addition of cAMP to wild-type cells, while the present study shows that growth arrest becomes appeared by exogenous addition of cAMP to cAMP-depleted cells. Given these differences in their stress responses, the mechanism underlying growth arrest induced by glucose limitation would be essentially distinct from that induced by salt or oxidative stress.

In addition to its involvement in sugar metabolism, cAMP-CRP has been shown to be more widely involved in various kinds of cellular functions, including flagellum biosynthesis, biofilm formation, virulence and persistence. cAMP-CRP participates in general stress response through activation of σ^S^ transcription, as described above. cAMP-CRP can directly induce transcription of stress-responsive genes by binding to the promoter sequence, as exemplified by *E. coli proP*, a transporter gene for the osmoprotectant proline (Landis et al., 1999). The present study demonstrates that cAMP-CRP is responsible for stress-induced growth arrest, which is a novel role for cAMP.

Recent study has suggested hundreds of promoter sequences on the *E. coli* genome involved in regulation by cAMP-CRP complex (Shimada et al., 2011). Nevertheless, little evidence is known that cAMP is involved in DNA replication, although there is one: Binding of cAMP to the replication initiator protein DnaA has been shown to promote rapid reactivation of inactive ADP-bound DnaA via removal of ADP (Hughes et al., 1988). This event is unusual in that the action of cAMP does not involve CRP and therefore may not be related to the stress-induced growth arrest observed in the present study.

Eukaryotic cells have a p53-based cellular regulatory system, which induces cell growth arrest in response to stress, and this system is independent of cAMP (Hafner et al., 2019). In the present study, we demonstrate that bacterial cells also have a system that induces growth arrest upon stress, and it is capable of quick response.

## Data Availability

The original contributions presented in the study are included in the article/Supplementary material, further inquiries can be directed to the corresponding author.
