# Supplementary material for: cAMP-CRP-activated E. coli causes growth arrest under stress conditions
Source: Front Microbiol. 2025 Aug 29;16:1597530. doi: 10.3389/fmicb.2025.1597530 (PMC12426135; doi:10.3389/fmicb.2025.1597530)
Supplement: Supplementary file 1 [file Supplementary_file_1.docx]

**Supplementary Materials**

**Figure S1. Growth rate.** Specific growth rates of wild-type, ∆*cyaA* and ∆*crp* cells were plotted every 10 min for up to 6 h. Measurement starts from the beginning of the main culture. At least three independent experiments were performed for each culture condition. Error bars represent the standard deviation.

**Figure S2. Growth curve when stress is introduced at OD_600_=0.5.** Growth curve when stress is introduced at OD_600_=0.5. Growths of wild-type, *∆cyaA* and *∆crp* cells were monitored with the addition of 0.9 M NaCl (A), 70 mM H_2_O_2_ (B) and 20 µg/ml plumbagin (C) at OD_600_=0.5. At least three independent experiments were performed for each culture condition. Error bars represent the standard deviation.

**Figure S3. Time course of CFU after stress.** The CFU values were overlaid on the growth curve of OD_600_ with the addition of 0.9 M NaCl (A), 70 mM H_2_O_2_ (B) and 20 µg/ml plumbagin (C). At least three independent experiments were performed for each culture condition. Error bars represent the standard error.

**Figure S4. OD_600_ values and CFU of *E. coli* cells at later stages after stress.** OD_600_ value and CFU of cells cultured without (A) and with the addition of 0.9 M NaCl (B), 70 mM H_2_O_2_ (C) or 20 µg/ml plumbagin (D) was manually measured after 10-fold dilution with LB medium. At least three independent experiments were performed for each culture condition. Error bars represent the standard error.

**Figure S5. Microscopic observation of cells.** One hour after stress with NaCl, H_2_O_2_ or plumbagin, 300 μl of cell culture was collected and heated at 85°C, followed by washing three times with PBS buffer. The cells were dissolved in 10 μl of double-distilled water, placed onto a microscope slide, and allowed to air dry. Subsequently, 10 μl of 90% ethanol was added to the sample and allowed to dry completely. This ethanol application and drying procedure was repeated two more times for proper fixation. After the final drying, 4', 6-diamidino-2-phenylindole dihydrochloride (DAPI, 1:1000 dilution; Gaithersburg, Maryland, USA) solution was added to the sample and incubated for 15 min. Imaging was performed using a confocal FV3000 (Evident) microscope equipped with a 60x objective lens, set at 500 HV, with a laser transmission of 3%, and a resolution of 4000 × 3200 dpi.

**Figure S6. Exogenous addition of various concentrations of cAMP** **to ∆*cyaA* cells.** *∆cyaA* cells were cultured with the exogenous addition of 3, 5, and 10 mM of cAMP at OD_600_=0. 0.9 M of NaCl (A), 70 mM of H_2_O_2_ (B) or 20 µg/ml plumbagin (C) was added at OD_600_=0.7 to introduce stress. At least three independent experiments were performed for each culture condition. Error bars represent the standard deviation.

**Methods**

**Measurement of Colony Forming Unit (CFU)**

CFU counting was performed under both non-stress and stress conditions, for salt and oxidative stress. When the culture reached OD_600_=0.7, stress inducers were added at the following final concentrations: 0.9 M NaCl, 70 mM H₂O₂, and 20 µl/ml plumbagin. Non-stressed samples were collected at OD_600_=0.7, while stress-induced samples were collected at 1-hour intervals after stress until recovery from growth arrest. At each time point, 100 µL of culture was taken and serially diluted up to 10⁷-fold. 100 µl of the diluted sample was spread onto LB agar plates that had been sprayed with sterilized water. Then, 0.5% soft agar was layered on the top. The plates were incubated at 37°C for 24 h, after which colonies were counted. The CFU values were overlaid on the time course of OD_600_.
