# Supplementary figures and images for: cAMP-CRP-activated E. coli causes growth arrest under stress conditions
Source: Front Microbiol. 2025 Aug 29;16:1597530. doi: 10.3389/fmicb.2025.1597530 (PMC12426135; doi:10.3389/fmicb.2025.1597530)

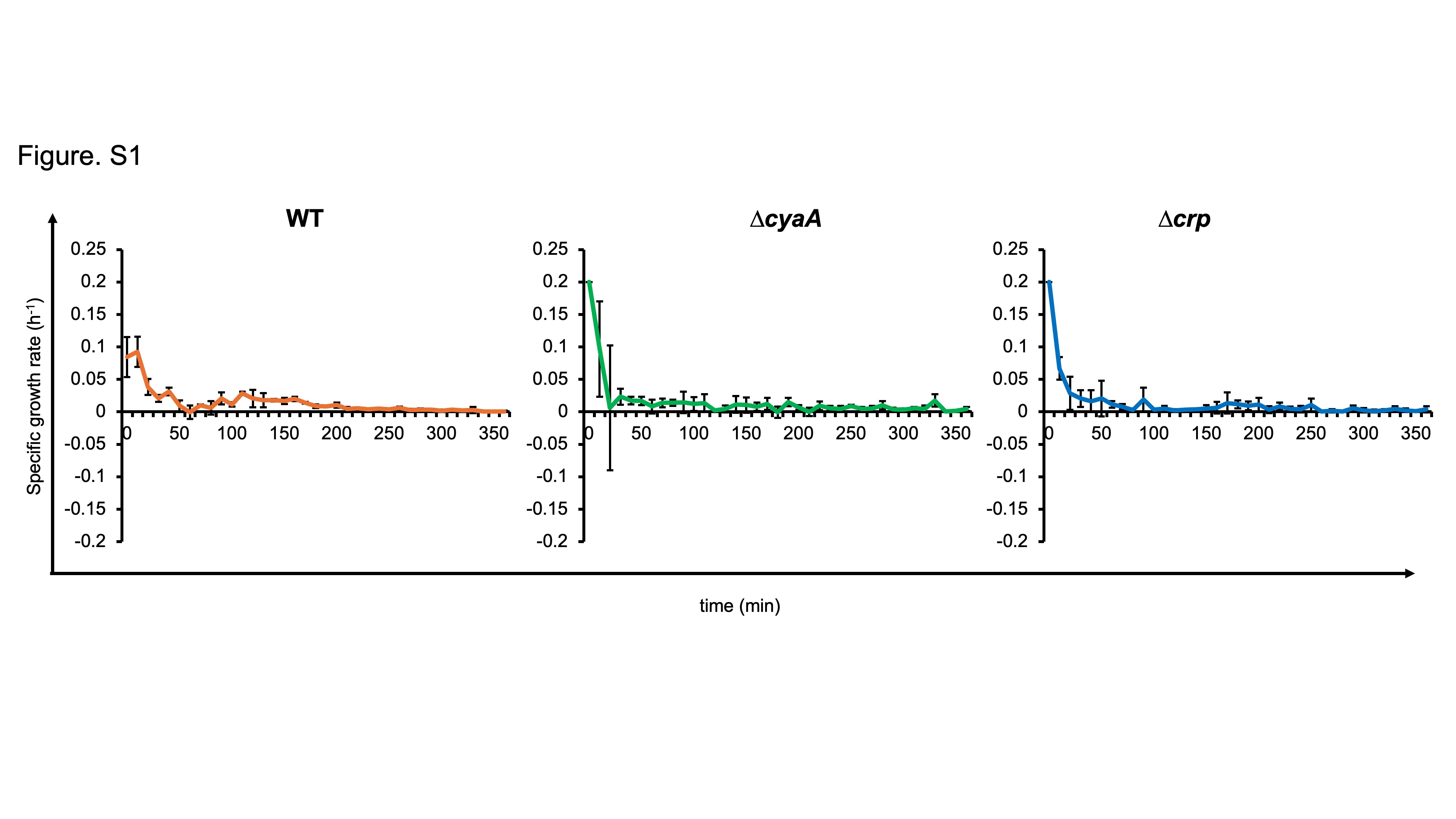

Supplement: Supplementary file 2 [file Image_1.jpeg]

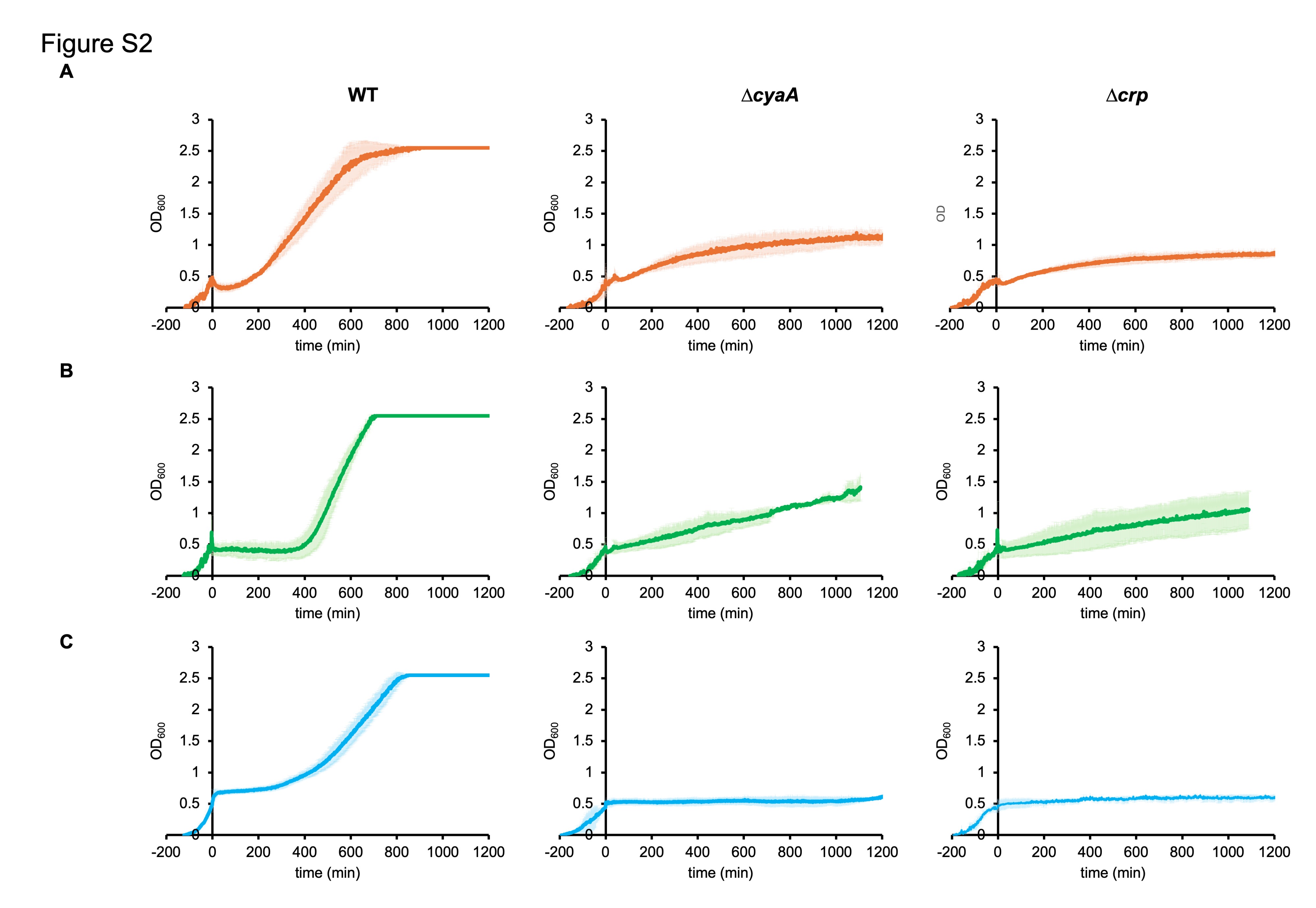

Supplement: Supplementary file 3 [file Image_2.jpeg]

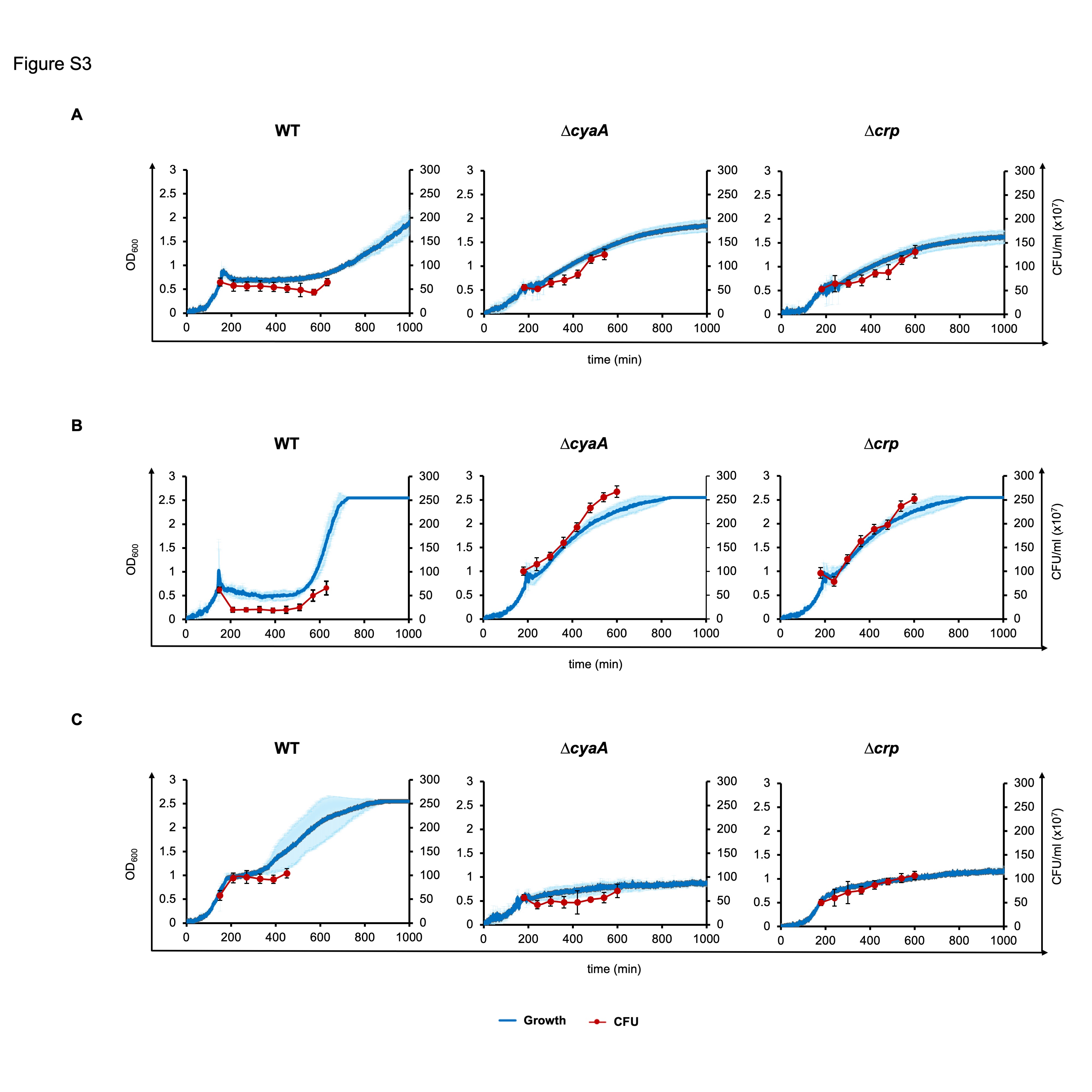

Supplement: Supplementary file 4 [file Image_3.jpeg]

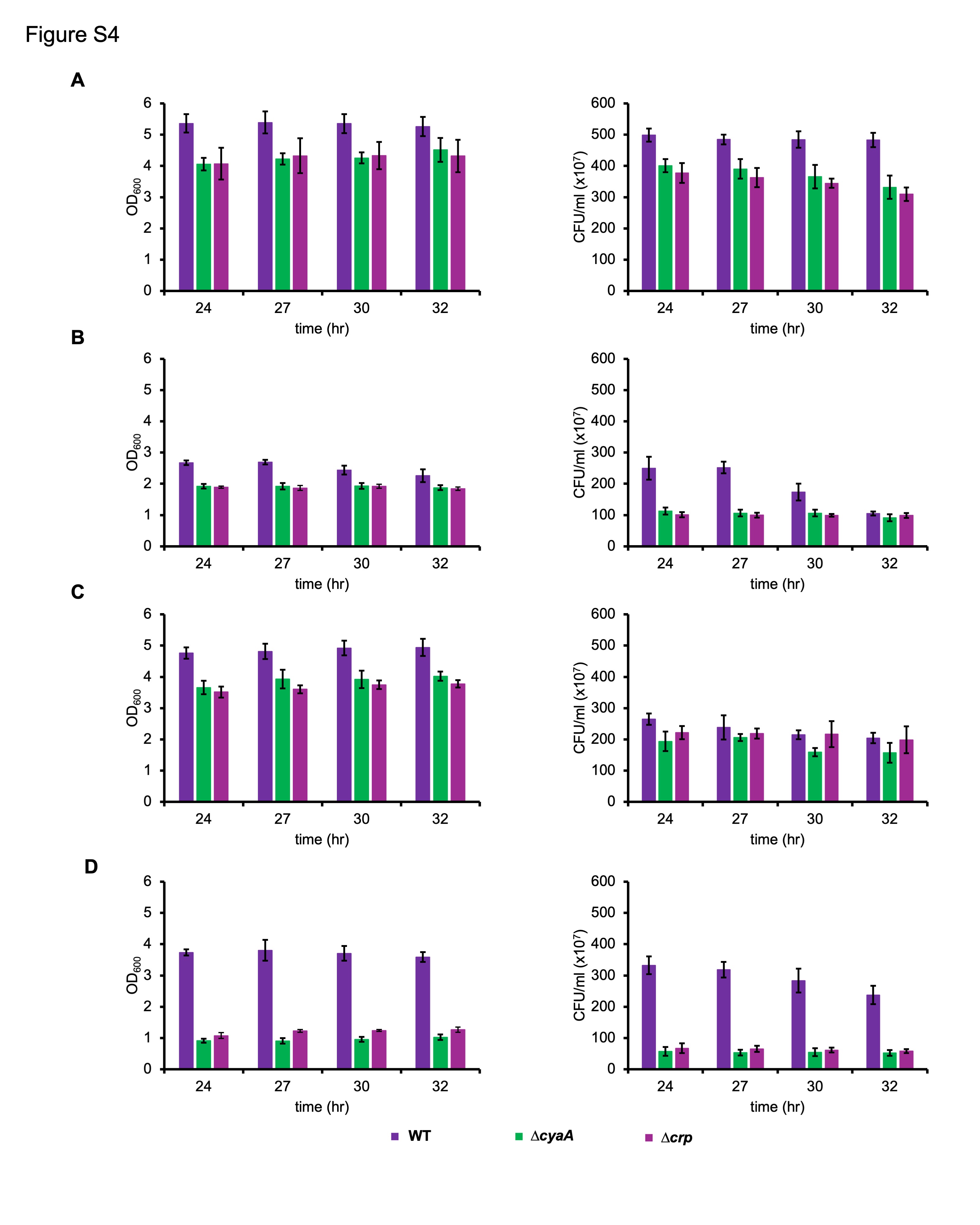

Supplement: Supplementary file 5 [file Image_4.jpeg]

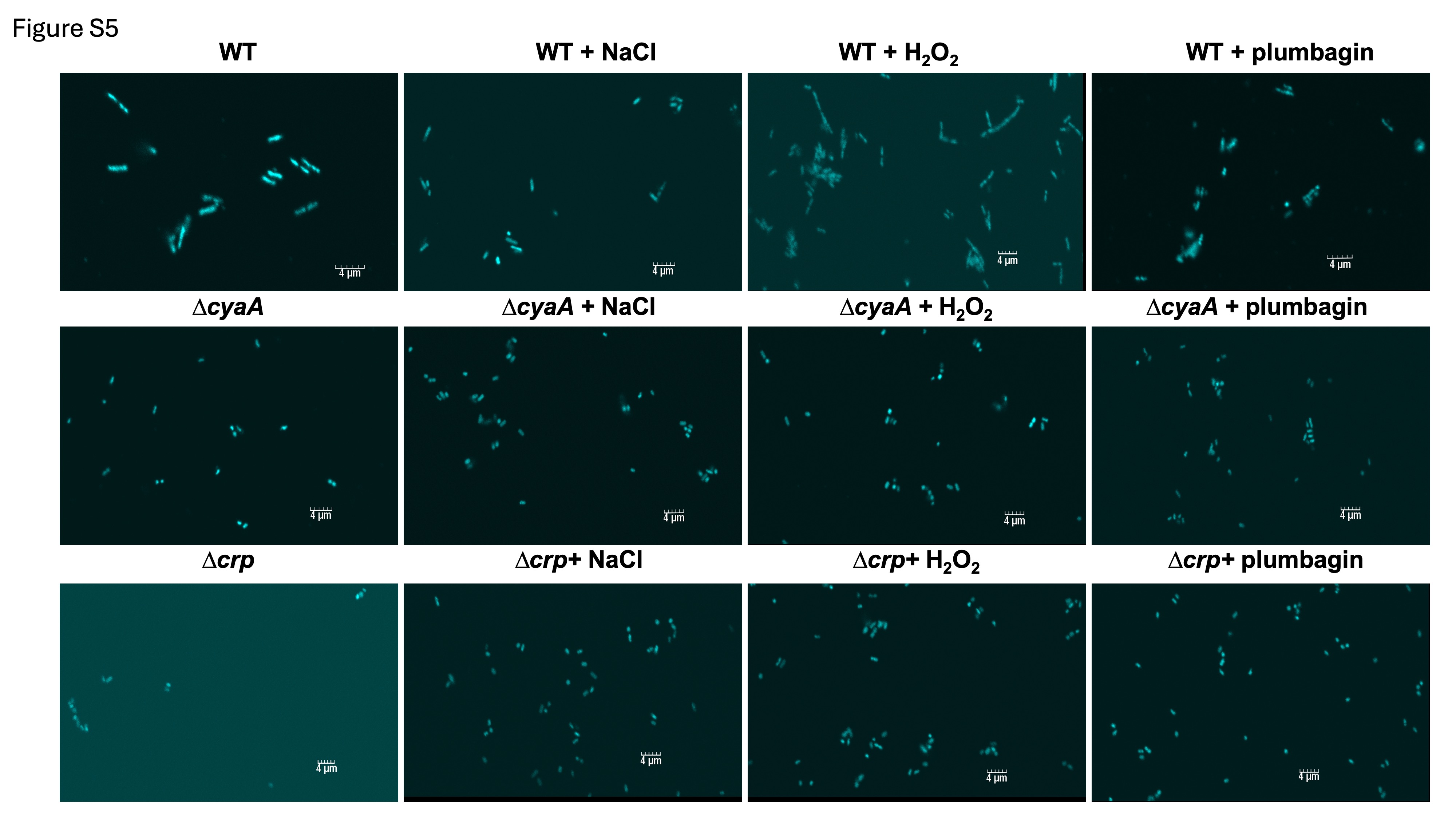

Supplement: Supplementary file 6 [file Image_5.jpeg]

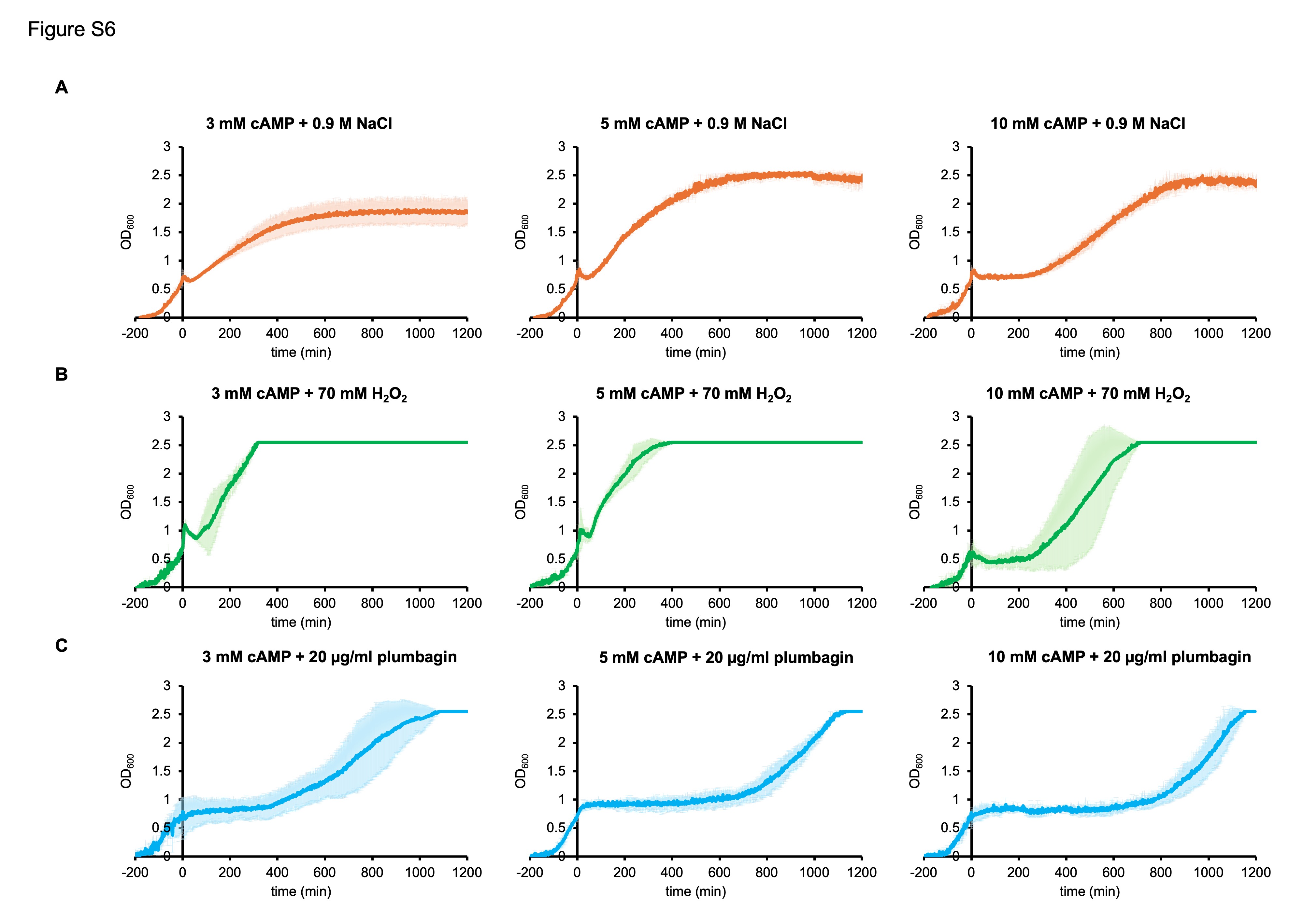

Supplement: Supplementary file 7 [file Image_6.jpeg]
